# Preliminary Blood Pressure Screening in a Representative Sample of Extremely Obese Kuwaiti Adolescents

**DOI:** 10.1155/2013/968754

**Published:** 2013-11-18

**Authors:** Rima Abdul Razzak, Asma Elmteri, Taiba Elkanderi, Fareedah Ishaq, Munera Eljasem, Saja AlDuraie, Jenan Al-Boloushi, Fatma Elbanay, Latifa Eldabos

**Affiliations:** ^1^Department of Physiology, Arabian Gulf University (AGU), Road 2904, Building 293, 329 Manama, Bahrain; ^2^CMMS, Arabian Gulf University (AGU), Road 2904, Building 293, 329 Manama, Bahrain

## Abstract

A relationship between blood pressure (BP) and obesity has been found in young adults, but no data are available for adolescents in Kuwait. 257 adolescent (11–19 years) participants were categorized into two groups according to their BMI; 48 nonobese (21 males: 43.7% and 27 females: 56.3%) with mean age of 15.61 ± 2.40 years and 209 obese (128 males: 61.25% and 81 females: 38.75%) with mean age of 15.02 ± 2.82 years. The mean BMI was 21.7 ± 2.23 kg/m^2^ for the nonobese group and 34.47 ± 4.70 kg/m^3^ for the obese group. Most BP measures based on a single screening were significantly higher in the obese group. The prevalence of elevated BP was significantly higher in the obese subjects (nonobese: 13%; obese: 63%; *P* < 0.0001). In the obese group, there was a significant positive correlation between total sample BMI and all BP measures except the pulse pressure. There was a similar rate of elevated blood pressure between males and females (64% versus 60%; *P* = 0.66). For both isolated systolic elevated BP and isolated diastolic elevated BP, the prevalences were comparable between the males (systolic: 42%; diastolic: 5%) and females (systolic: 34%; diastolic: 14%). Only systolic BP was positively correlated with BMI in obese adolescent males (Spearman *r* = 0.18; *P* < 0.05), with a significant correlation between BMI with diastolic (Spearman *r* = 0.22; *P* < 0.05) and mean BP (Spearman *r* = 0.21; *P* < 0.05) in females.

## 1. Introduction

Clinical studies have already confirmed a strong relationship between obesity and hypertension [1] with visceral obesity seeming to represent the most important risk factor for hypertension and cardiovascular disease [2]. Although the exact mechanism of how obesity is a cause of hypertension is unknown, many theories have been suggested such as activation of the renin-angiotensin-aldosterone system, oxidative stress, sympathetic overdrive, chronic vascular inflammation and endothelial dysfunction, which leads to structural changes such as thickening of the intima and media of the vessel wall [3, 4]. Obesity is an increasing worldwide health problem phenomenon especially in adolescents, and it is one of the main factors, in addition to family history, contributing to the increase in the prevalence and rate of diagnosis of hypertension in children and adolescents [5], with primary hypertension being very common in adolescents [6]. The consequence of adolescent obesity was reported in a study which showed that BMI greater than the 75th percentile in adolescence usually lead to an increased risk of death from cardiovascular disease [7] in adulthood. 

Many studies have emphasized the increasing obesity among adolescents in Kuwait. A study carried out in 2005 on adolescent Kuwaiti girls showed that they rank the highest in obesity compared with the same sex and age group in Egypt and Lebanon [8]. More recently Musaiger et al. [9] showed that in comparison to other Arabic states, Kuwaiti adolescents showed the highest prevalence of obesity for both males (34.8%) and females (20.6%). This extends to Kuwaiti adolescents being among the highest in obesity prevalence not only in the Gulf countries but in the world reaching up to 40–46% [10, 11]. This was attributed to the rapid socio-economic growth in Kuwait and Arabian Gulf states in general, with the availability and consumption of fast food increasing by adolescent individuals.

In Kuwait, the prevalence of hypertension in young and middle aged Kuwaiti citizens of a minimum age of 21 years [12] was determined, with the study emphasizing the influence of body mass index on blood pressure and a major lack of previous knowledge or awareness of hypertension in hypertensive individuals. Surprisingly, in spite of the high prevalence of obesity in Kuwaiti adolescents, data on the prevalence of elevated blood pressure or hypertension in obese Kuwaiti adolescents does not exist. Accordingly, the purpose of this study was (1) to preliminarily estimate the prevalence of elevated BP in a representative small sample of extremely obese Kuwaiti adolescents and (2) to investigate the relationship between BP and body mass index (BMI) based on a single screening. As there is inconsistent or regular blood pressure monitoring in children and adolescents during visits to health centers in Kuwait, the importance of this study is to emphasize the significance of early detection of hypertension and intervention in adolescents with hypertension in Kuwait.

## 2. Materials and Methods

This study took place in Kuwait, a member of the Arabian Gulf states. Data for this study was collected between July and September, 2012. 257 participants of different adolescent ages (11–19 years) were selected on a volunteer-basis from Kuwait University, Capacity Academy courses, Kuwait Science Club (KSC), the Scientific Center, Saad Club, AL-Habara Club, Health Center and Shaab Leisure Park. The selection of subjects was based on examiners' subjective estimation of obesity by sight, and to avoid embarrassment to the obese volunteers, some non-obese subjects were also asked to volunteer. Information about the participants' age, gender, family history, smoking, alcohol, diet habits, exercise, cardiovascular disease and previous history of hypertension was also taken.

Before blood pressure (BP) measurement, the participants were asked about their intake of any stimulants. The weight and height of the participants were measured in a standing position (bare feet) using a precision electronic scale (HD-313 Digital Weight Scale, Tanita) and a stadiometer (Seca 222, Seca) respectively. The weight was measured in kilograms (kg) and the height in centimeters (cm). After measuring the weight and height the body mass index (BMI) was calculated as weight divided by height squared (kg/m^2^). BMI has been recommended as the most acceptable, valid and reproducible measure of body fat in children and adolescents which is both valid and reproducible [13]. In normal children, BMI increases slightly with age, thus BMI percentiles, which are age specific, are used to define risk categories [14, 15], and BMI criteria of the International Obesity task Force (IOTF) were used to define obesity. The IOTF classification system provides extended BMI cut-points by age and sex for overweight, obesity, and severe obesity among children aged 2–18 years [16]. Subjects with a BMI >30 kg/m^2^ were categorized as obese or severely obese according to the extended BMI cut-points. In this study, they would only be referred to as obese subjects. As the main aim of this study was to estimate the prevalence of elevated BP in a representative small sample of extremely obese Kuwaiti adolescents, the sample size of non-obese subjects in this study was much smaller (*n* = 48; 27 females: 56.3% and 21 males: 43.7%) than the obese sample (*n* = 209; 81 females: 38.75% and 128 males: 61.25%). The subjects in the severely obese group were divided into three adolescence stages, but this was not carried out for the non-obese group due to the small sample size. 

An oscillometric fully-automated wrist device (BP W100, Microlife, *Switzerland*) was used to measure BP; the device had a blood pressure measurement range of 30–280 mm Hg and a statistic accuracy within ±3 mm Hg. As designated on the device, it was clinically validated according to the European Society of Hypertension (ESH) and the British Hypertension Society (BSH) protocols that require that automated device be compared with blood pressures measured by trained observers using a mercury sphygmomanometer and stethoscope. The width of the cuff was adjusted to the wrist circumference, and BP was measured in a comfortable sitting position two times on the right wrist with the arm supported and the cuff at the level of the heart, with 1 minute interval between the measurements. The average systolic and diastolic pressures were used to define BP. Percentiles of systolic and diastolic BP was generated using the World Health Organization (WHO) reference tables for Blood Pressure Levels by Age and Height Percentile. BP was diagnosed according to the official guidelines issued by the American Heart Association. Even though the new guidelines recommend multiple BP measurements at different sessions to define persistent hypertension and pre-hypertension, BP was measured two times in only one session in the present study. Accordingly, we adopted Din-Dzietham et al. [17] definition of high (elevated) blood pressure instead of hypertension however the same cutoff points of classification were used (Table 1). Elevated or high BP was considered for systolic and/or diastolic pressures >95th percentile for age.

Data was analyzed using the GraphPad InStat3 software. Descriptive statistics were presented as means, standard deviations (SDs) and percentages. The Kolmogorov and Smirnov test was used to assess the normality of the data. Comparison of means was carried out by the appropriate statistical test: two-sample Student's *t* tests were performed for between group comparisons of continuous variables with a normal distribution, and the Mann-Whitney test on variables with non-normal distributions. Spearman's rho was used for testing statistical dependence between two variables. For treatment of frequencies, the Fischer's Exact test was used. For all tests, the significance level was fixed at 0.05.

## 3. Results

The mean age was 15.61 ± 2.40 years for the non-obese subjects and 15.02 ± 2.82 years for the obese subjects. The BMI values in the non-obese group followed a Gaussian distribution. In the obese group, the BMI values of the whole sample and some of the different age groups did not follow a normal distribution. Accordingly the mean was reported but the median was used for statistical comparison. 

The mean BMI for the non-obese group was 21.7 ± 2.23 kg/m^2^, with no difference between sexes (male: mean BMI = 22.31 ± 2.25 kg/m^2^; female: mean BMI = 21.30 ± 2.22 kg/m^2^, *P* = 0.14). Comparison as in Table 2 between the obese and non-obese subjects shows very significantly larger values of BMI in the obese sample. 

Table 3 also illustrates the mean BMI values for each sex at different stages of adolescence. Clearly, the mean BMI value is greater than 30 kg/m^2^ for both sexes and coincides with cut-off values for severe obesity according to ITFO. There was no significant difference at any adolescent stage when comparing with the Mann-Whitney Test the median values for males and females (Median: males: 33.90, females, 33.00; *P* = 0.21). 

BP values in Tables 2 and 3 represent measurement of a single screening. Comparison in blood pressure parameters between the non-obese and obese group shows that the systolic BP and MAP in the obese subjects were very significantly larger than the non-obese group, with the diastolic BP marginally showing a difference (Table 2). 

Table 3 values focus on BP measures from the obese subjects at three adolescent stages. It is obvious that there was a progressive increase in blood pressure measures with adolescence age, and values of all BP measures were highest for the age group of 17−19 years in both sexes. For systolic BP, the range in obese males was 102–179.5 mm Hg, and in obese females was 102–158 mm Hg, with the mean being significantly greater in males than females by almost 7 mmHg (*t* = 2.16, *P* = 0.03) in the middle adolescent stage and 8.5 mm Hg (*t* = 2.89, *P* = 0.005) in late adolescent stages. For diastolic BP, the range in males was 59–138 mm Hg, and in females was 50–129 mm Hg. Even though there was no statistical significance, it is worth pointing out that it was only in the late adolescent stage the diastolic pressure in males was about 6 mm Hg higher than in females.

As for the frequency of subjects with high BP in each group, Tables 2 and 3 show that 133 (63%) obese subjects had elevated BP on this initial screening according to the classification of the American Heart Association; there were only 6 subjects with elevated BP n the non-obese sample, representing a significantly less percentage than in the obese group (13% versus 63%; Fisher's Exact test, *P* < 0.0001).

In the obese group, males as compared to females, had a non-significantly higher rate of elevated BP on first screening (64% versus 60%; Fisher's Exact test, *P* = 0.66) however the spread along adolescence years was different in both sexes (Table 3). In males the percentage of subjects with elevated BP increased with increasing adolescence stage, and an opposite pattern was evident in the females. In males there was an increase of 9% prevalence between early adolescence and middle adolescence, and there was a 12% increase between middle adolescence and late adolescence. In females, there was a decrease of 8% prevalence between early adolescence and middle adolescence and 6% between middle adolescence and late adolescence.

**Figure 1:**
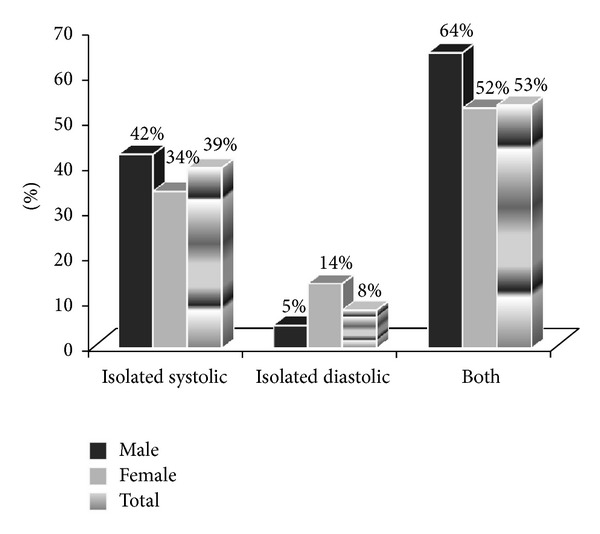
Frequency of types of elevated blood pressures among the obese Kuwaiti adolescents.

In the 133 obese subjects with elevated BP, the type of elevated BP which was contributing to the elevated BP was evaluated by frequency plot (Figure 1). The figure shows that more than half (70/133; 53%) of the obese subjects had both elevated systolic and diastolic BP. Males contributed more to the high isolated systolic BP (35/83; 42%) than females (17/50; 34%) but non-significantly (Fisher's Exact test, *P* = 0.37), while the opposite was true for females who non-significantly contributed more to the high isolated diastolic BP (7/50; 14%) compared to males (4/83; 5%) (Fisher's Exact test, *P* = 0.10).

Spearman correlation analyses between body mass index (BMI) and different measures of BP show that BMI of the total obese sample was significantly and positively correlated with systolic, diastolic and very significantly with the mean arterial pressure (MAP), but with no association with the pulse pressure (Table 4). Analysis further shows that in males, it was only the systolic BP that was significantly related to BMI, while in females both the diastolic BP and MAP were significantly correlated to BMI. 

## 4. Discussion and Conclusion

The current study emphasizes many major findings relating obesity and hypertension in adolescents in Kuwait. Due to inconsistencies in working definitions of severe obesity, we adopted the newly published IOTF cut-points of obesity and extreme obesity, because they are now recommended for international use [16]. Accordingly, all our obese adolescent subjects are considered obese to extremely obese, since the minimum BMI was 30 kg/m^2^ for both sexes and the range was up to 56.4 kg/m^2^. 

In the obese sample, it is clear that the BP values were increasing with age, with minimal difference between the early stages of adolescence in the mean systolic BP in females, but occurring at later stages. The significant difference in systolic BP between males and females in mid and late adolescence stages confirm the fact that sexual dimorphism in arterial blood pressure appears in adolescence. This dimorphism persists throughout adulthood [18] up to 60 years of age, with average systolic and diastolic pressures in men being higher than aged-matched females by 6-7 and 3–5 mm Hg respectively [19]. This may be attributed to the blood pressure lowering effect of estradiol in women and in our study to the possibility that menstrual regularity and sex hormone levels are still not well established in early adolescence. 

It is also evident from the results that there was a high rate of elevated BP in the obese adolescent group. The association of the presence of obesity with much higher rates of hypertension has been previously established in other countries. In the report by McNiece et al. [20] on high school students, the prevalence of hypertension and pre-hypertension combined was more than 30% in obese adolescent boys and 23–30% in obese adolescent girls depending on ethnicity. Another study by Sorof et al. [5] reported a hypertension prevalence of 10.7% in multi-ethnic adolescents with BMI ≥95th percentile after 3 screenings. In our subjects, systolic, diastolic and mean BP was strongly associated with the BMI in the total obese sample. Systolic BP, diastolic BP and mean BP all increased with BMI. The high rate of isolated systolic high BP (39%), as well as elevated systolic along with elevated diastolic blood pressure (53%) in the obese adolescents in this study is alarming. In adults, isolated systolic hypertension was found to pose a strong and independent risk of cardiovascular mortality [21], with isolated systolic hypertension being more associated than isolated diastolic hypertension with risk of coronary heart disease, stroke and end-stage renal disease [22]. A relatively recent systematic review and meta-regression analysis study that examined the tracking of blood pressure (BP) from childhood to adulthood has confirmed that childhood BP is associated with BP in later life [23].

There are a number of shortcomings in our study. Initially, the size of the non-obese sample was small and may not be an appropriate representation of the non-obese Kuwaiti adolescent population. However, the main aim of this paper was to focus on obese subjects and to estimate the prevalence of elevated BP in this representative group. An additional limitation is the high prevalence of elevated BP in the obese sample, and this could be attributed to methodological factors. An important factor is our measurement of BP on one single occasion. The new guidelines recommend multiple BP measurements at different sessions to define persistent hypertension and prehypertension [24]. This is because it was previously shown that the number of subjects showing elevated BP values at the first measurement was double or more the number of subjects having BP measurement on two or three different visits [5]. In Sorof's study on school-aged children, the prevalence of elevated blood pressure after first, second and third screenings was 19.4%, 9.5% and 4.5% respectively. Following this rational, this implies that if additional screening measurements had been performed on subsequent occasions in the current study, it is almost certain that the overall prevalence of elevated BP would have continued to decrease probably to 30% on the second screening. Nevertheless, elevated single BP measurements should not be undermined, because 68% of adolescent boys and 43% of adolescent girls diagnosed with prehypertension and hypertension combined, on the basis of a single BP measurement, had prehypertension or hypertension 2 years later [25].

Another methodological factor for the high incidence of elevated BP could be the use of the automated oscillometric wrist BP monitor instead of the upper arm monitor. There has been much debate in recent years over the clinical accuracy of wrist BP monitors. A recent study led by Doshi et al. [26] have shown that even when a validated wrist device was used for BP measurement in a standardized protocol, there were significant differences for systolic BP and diastolic BP measured at the arm and wrist. In their study on obese patients, they found higher wrist values, with the majority of patients having systolic BP or diastolic BP absolute differences of ≥5 mm Hg between the two methods, and approximately one third having a difference of ≥10 mm Hg in systolic BP and diastolic BP. 

In conclusion, the low degree of awareness among Kuwaitis of the risks of adolescent obesity has to be rectified in the light of the risk of the rapid socioeconomic growth in Kuwait. Actions such as increasing awareness programs in schools in Kuwait as well as continuous monitoring of blood pressure in children and adolescents during health center visits have to be emphasized and implemented further in Kuwait.

## Figures and Tables

**Table 1:** Classification of hypertension in adolescents.

Blood pressure classification	Adolescents younger than 18 y	Adolescents 18 y and older
Normal	SBP and DBP <90th%	SBP <120 mm Hg and DBP <80 mm Hg
Prehypertension	SBP or DBP 90th–95th%; or if BP is >120/80 mm Hg even if <90th%	SBP 120–139 mm Hg or DBP 80–89 mm Hg
Stage 1 hypertension	SBP or DBP ≥95th–99th% plus 5 mm Hg	SBP 140–159 mm Hg or DBP 90–99 mm Hg
Stage 2 hypertension	SBP or DBP >99th% plus 5 mm Hg	SBP ≥160 mm Hg or DBP ≥100 mm Hg

DBP: diastolic blood pressure; SBP: systolic blood pressure. Adapted with permission [20, 24].

**Table 2:** Comparison of BMI and blood pressure parameters between non-obese and obese Kuwaiti adolescents (11–19 years of age).

	BMI (kg/m^2^)	Systolic BP (mm Hg)	Diastolic BP (mm Hg)	MAP (mm Hg)	Elevated BP *N* (%)
Non obese (*n* = 48)	21.7 (2.23) (17.0–24.7)	119.9 (13.4) (102.5–142.0)	77.1 (13.0) (52.0–115.0)	90.5 (9.8) (67.7–109)	6 (13%)
Obese (*n* = 209)	34.5 (4.7) (30.1–56.4)	131.6 (14.7) (102.0–179.5)	81.3 (13.8) (50.0–138.5)	97.9 (13.1) (72.8–150.5)	133 (63%)
*P *	<0.0001	<0.0001	0.05	0.001	<0.0001

BMI: body mass index; BP: blood pressure; MAP: mean arterial blood pressure.

Values are means (S.D).

Elevated BP: defined as systolic and/or diastolic pressures >95th percentile for age.

**Table 3:** Mean age, BMI, and incidence of elevated BP among obese Kuwaiti adolescents, by sex and different adolescence stages.

Sex	Age (year)	*N *	BMI (kg/m^2^)	Systolic BP (mm Hg)	Diastolic BP (mm Hg)	MAP (mm Hg)	Elevated BP *N* (%)
Males	11–13	48	33.8 (3.9)	124.4 (13.2)	76.0 (12.7)	92.2 (12.0)	25 (52%)
	14–16	38	35.2 (4.8)	132.7 (12.2)	78.1 (9.3)	96.3 (9.4)	23 (61%)
	17–19	42	35.1 (4.5)	142.9 (12.8)	91.8 (12.8)	108.8 (11.6)	35 (83%)
	All	128	34.6 (4.4)	132.9 (14.8)	81.8 (13.7)	98.8 (13.2)	83 (64%)

Females	11–13	20	32.2 (3.4)	125.6 (10.8)	74.9 (12.4)	91.7 (11.5)	14 (70%)
	14–16	29	35.4 (6.1)	125.8 (13.6)*	78.0 (9.9)	93.8 (9.0)	18 (62%)
	17–19	32	34.3 (5.0)	133.4 (15.3)**	85.5 (16.0)	101.5 (14.9)*	18 (56%)
	All	81	34.2 (5.2)	128.8 (14.1)*	80.1 (14.1)	96.3 (12.8)	50 (60%)

Both	Total	209	34.47 (4.7)	131.6 (14.7)	81.3 (13.8)	97.9 (13.1)	133 (63%)

BMI: body mass index; BP: blood pressure; MAP: mean arterial blood pressure.

Values are means (SD).

Elevated BP: defined as systolic and/or diastolic pressures >95th percentile for age.

Difference between male and female; *significance at *P* < 0.05; **significance at *P* < 0.01.

**Table 4:** Spearman correlation coefficients between BMI and different measures of blood pressure in obese Kuwaiti adolescents.

Systolic BP			Diastolic BP			MAP			Pulse P		
Both	M	F	Both	M	F	Both	M	F	Both	M	F
0.18*	0.18*	0.14	0.16*	0.10	0.22*	0.18**	0.14	0.21*	0.03	0.07	0.09

Correlation between BMI and blood pressure measure, *significance at *P* < 0.05; **significance at *P* < 0.01.

BP: blood pressure; MAP: mean arterial pressure.

M: male; F: female.
